# Clinicogenetic correlations in behavioral variant frontotemporal dementia

**DOI:** 10.1002/alz70855_106845

**Published:** 2025-12-25

**Authors:** Allison Snyder, EunRan R Suh, Laynie Dratch, Lauren Massimo, Eddie B. Lee, David J. Irwin, Vivianna M Van Deerlin, Corey T. McMillan

**Affiliations:** ^1^ Penn Frontotemporal Degeneration Center, Department of Neurology, Perelman School of Medicine, University of Pennsylvania, Philadelphia, PA, USA; ^2^ Center for Neurodegenerative Disease Research, Perelman School of Medicine, University of Pennsylvania, Philadelphia, PA, USA; ^3^ University of Pennsylvania, Philadelphia, PA, USA; ^4^ Department of Pathology & Laboratory Medicine, Perelman School of Medicine, University of Pennsylvania, Philadelphia, PA, USA; ^5^ Center for Neurodegenerative Disease Research, Department of Pathology and Laboratory Medicine, Perelman School of Medicine, University of Pennsylvania, Philadelphia, PA, USA

## Abstract

**Background:**

Behavioral variant frontotemporal dementia (bvFTD) is the most common clinical presentation of FTD with considerable genetic and neuropathological heterogeneity. Here, we characterize a large cohort (*n* = 410) at Penn to gain new insights into bvFTD pathology and genetics in light of advances in our understanding of disease.

**Method:**

Individuals meeting Rascovsky bvFTD criteria were included; individuals with primary ADNC or LBD were excluded. We characterize familial risk via 3‐generation pedigrees by Wood Criteria, report known monogenic variants, and perform a gene burden analysis to evaluate the impact of rare variants on bvFTD. We also characterize neuropathological features in a subset of individuals (*n* = 88).

**Result:**

410 bvFTD cases were identified, 56.3% male; mean age was 62.6 years (+/‐ 7.8); mean CDR FTLD SB was 9.0 (+/‐ 3.7). Wood's risk scoring identified 21.4% apparent sporadic; 17.8% high; 11.4% medium; 14.6% low; and 21.7% unknown familial risk, yielding a prediction of 74 genetic cases within the cohort; 107 monogenic cases were identified. In available neuropathologic data (*n* = 88), 59.1% were FTLD‐TDP, 39.8% FTLD‐Tau, and 1.1% FTLD‐FET. The most common secondary pathologies were ADNC (27.3%), no pathology (25%), and secondary FTLD pathology (22.7%). Secondary ADNC was slightly higher in FTLD‐Tau (31.4%) vs. FTLD‐TDP (25%); rates of any ADNC was 37% in FTLD‐Tau and 50% in FTLD‐TDP. There were 6 *APOE* e4/e4 carriers in the autopsy cohort, but none with ADNC. The *MAPT* H1/H1 haplotype was more prevalent in FTLD‐Tau (77.8%) vs. FTLD‐TDP (62%).

**Conclusion:**

Our findings are consistent with previous reports indicating approximately 60% of bvFTD cases are FTLD‐TDP with slight male predominance. Surprisingly, we found high rates of co‐pathology—specifically ADNC. This was more common in FTLD‐TDP than FTLD‐Tau and not associated with *APOE* e4 homozygosity. High rates of clinically relevant ADNC have increasing relevance because of newly‐available disease‐modifying therapies. We found a relative *MAPT* H1 risk haplotype enrichment in FTLD‐Tau compared to FTLD‐TDP. We identified a 44.6% greater‐than‐expected number of monogenic cases, suggesting that bvFTD is relatively enriched for familial forms compared to the full FTD spectrum. Future work will focus on gene burden analysis to identify rare variants that contribute to disease

